# Cognitive performance and active lifestyle in community-dwelling older adults

**DOI:** 10.1590/1980-5764-DN-2025-0431

**Published:** 2026-08-24

**Authors:** Gabriela dos Santos, Tiago Nascimento Ordonez, Beatriz Aparecida Ozello Gutierrez, Thais Bento Lima da Silva

**Affiliations:** 1 Universidade de São Paulo, Escola de Artes Ciências e Humanidades, Departamento de Gerontologia, São Paulo SP, Brazil. Universidade de São Paulo Escola de Artes Ciências e Humanidades Departamento de Gerontologia São Paulo SP Brazil; 2 Universidade Federal do Recôncavo da Bahia, Centro de Ciências da Saúde, Santo Antônio de Jesus BA, Brazil. Universidade Federal do Recôncavo da Bahia Centro de Ciências da Saúde Departamento de Gerontologia Santo Antônio de Jesus BA Brazil; 3 Universidade de São Paulo, Faculdade de Medicina, Grupo de Neurologia Cognitiva e do Comportamento, São Paulo SP, Brazil. Universidade de São Paulo Faculdade de Medicina Grupo de Neurologia Cognitiva e do Comportamento São Paulo SP Brazil

**Keywords:** Aged, COVID-19, Cognition, Social Participation, Idoso, COVID-19, Cognição, Participação Social

## Abstract

An active lifestyle promotes physical and mental health, improves social interaction, and provides cognitive stimulation, positively influencing mood and cognitive performance. However, lifestyle habits changed substantially after March 2020 due to the COVID-19 pandemic, particularly among high-risk groups such as older adults.

**Objective:**

To investigate the relationship between an active lifestyle and cognitive performance in community-dwelling older adults during the COVID-19 pandemic.

**Methods:**

A cross-sectional study of 50 older adults enrolled at a community center was conducted. Cognitive status was assessed using the Addenbrooke’s Cognitive Examination, whereas psychosocial indicators were assessed using the Control, Autonomy, Self-realization and Pleasure Scale, the Depression, Anxiety and Stress Scale, and the Geriatric Depression Scale. Leisure-time activities were assessed using the Brazilian Portuguese version of the Minnesota Leisure Time Activities Questionnaire, based on retrospective self-report of activities performed over the previous 12 months.

**Results:**

Participants with greater engagement in the activities investigated had better cognitive performance (p=0.021) and fewer depressive symptoms, although the difference was not statistically significant (p=0.060). Physical activity was associated with fewer anxiety symptoms (p=0.013) and greater autonomy (p=0.047). Engagement in intellectually stimulating activities was associated with better performance across all cognitive domains assessed.

**Conclusion:**

An active lifestyle was associated with better cognitive functioning and mental health in older adults.

## RESUMO

Um estilo de vida ativo promove a saúde física e mental, melhora a interação social e proporciona estimulação cognitiva, influenciando positivamente o humor e o desempenho cognitivo. No entanto, os hábitos de vida mudaram substancialmente após março de 2020 devido à pandemia de COVID-19, especialmente entre grupos de maior risco, como os idosos.

**Objetivo:**

Investigar a relação entre um estilo de vida ativo e o desempenho cognitivo em idosos residentes na comunidade durante a pandemia de COVID-19.

**Métodos:**

Foi realizado um estudo transversal com 50 idosos vinculados a um centro comunitário. O estado cognitivo foi avaliado por meio do Exame Cognitivo de Addenbrooke, enquanto os indicadores psicossociais foram avaliados pela Escala de Controle, Autonomia, Autorrealização e Prazer, pela Escala de Depressão, Ansiedade e Estresse e pela Escala de Depressão Geriátrica. As atividades de lazer foram avaliadas por meio de versão brasileira do *Minnesota Leisure Time Activities Questionnaire*, com base no autorrelato retrospectivo das atividades realizadas nos 12 meses anteriores.

**Resultados:**

Os participantes com maior engajamento nas atividades investigadas apresentaram melhor desempenho cognitivo (p=0,021) e menos sintomas depressivos, embora a diferença não tenha sido estatisticamente significativa (p=0,060). A atividade física foi associada a menos sintomas de ansiedade (p=0,013) e maior autonomia (p=0,047). O envolvimento em atividades intelectualmente estimulantes foi associado a melhor desempenho em todos os domínios cognitivos avaliados.

**Conclusão:**

Um estilo de vida ativo esteve associado a melhor funcionamento cognitivo e saúde mental em idosos.

## INTRODUCTION

Lifestyle is recognized as a determining factor of physical, mental, and cognitive health during the aging process and in later life. Staying active throughout the lifespan is associated with improved quality of life, fewer depressive and anxiety symptoms, and better cognitive performance^1,2^.

Despite public policies encouraging the adoption and maintenance of educational and leisure activities, among others, including for older adults, sociodemographic variables such as race, income, and education impact opportunities for social participation and an active lifestyle. Black, low-income, and low-educated populations tend to engage less in physical, educational, religious, civic, work-related, and leisure-time activities compared with White, higher-income, and more highly educated individuals^3,4,5^.

The neighborhood of residence in the city of São Paulo, for instance, represents a determinant of lifespan. Individuals living in the neighborhood of Moema, located in the southern part of the city, have a mean lifespan of 79.5 years, whereas those living in the district of Pedreira, also situated in the south, have a mean age at death of 62.7 years.

Approximately 15,000 older individuals reside in the district of Pedreira, of whom 47.3% are Black or Brown, 76.72% are illiterate or have incomplete primary education, 30% lack access to items deemed essential for well-being (*e.g*., a refrigerator, telephone line, or television), and 21.9% have some degree of functional disability^6^.

Public services are provided for populations with characteristics typical of residents of the district of Pedreira, *i.e*., group with greater social vulnerability, such as Community Centers for Older Adults. These public entities constitute primary social welfare services aimed at stimulating interaction and strengthening community ties.

The situation caused by the outbreak of the COVID-19 pandemic, beginning in March 2020, hampered the ability to maintain an active lifestyle, particularly among older adults, a high-risk group for COVID-19. Recent literature has shown that lockdown measures had a negative impact on cognitive performance and increased the prevalence of depressive and anxiety symptoms in older individuals^2,7,8^.

To date, few studies have reported the impacts of lockdown during the COVID-19 pandemic on cognitively healthy older adults based on pre- and post-pandemic cognitive assessments. De Pue et al.^9^ studied perceived cognitive decline reported by older individuals over three interview cycles conducted during different waves of COVID-19 in Belgium. The results showed greater perceived subjective cognitive decline among respondents during the follow-up period.

The present study can contribute to the literature investigating the maintenance of engagement in activities that promote an active lifestyle among cognitively healthy older adults during situations of social isolation, such as that caused by the COVID-19 pandemic. These results can help inform actions aimed at maintaining the physical, psychological, and cognitive health of these individuals, most of whom have few opportunities to lead an active and healthy later life.

Therefore, the objective of this study was to investigate the relationship between active lifestyle and cognitive performance in community-dwelling older adults.

## METHODS

### Participants

A total of 50 older adults were interviewed. Inclusion criteria were: having sufficient cognitive, visual, and motor abilities to complete the data collection instruments. Exclusion criteria were:

- individuals aged <60 years;
- older individuals with hearing, visual, or motor deficits precluding comprehension of the instructions and completion of the tasks; and
- a history of psychiatric disorders or a previous diagnosis of dementia.

The inclusion and exclusion criteria were applied based on participant self-reports.

### Instruments

Data were collected on sociodemographic, cognitive, and psychosocial variables, as well as aspects describing engagement in physical, domestic, leisure-time, cognitively stimulating, and work-related activities. Median scores for the variables measured using the Brazilian Portuguese version of the Minnesota Leisure Time Activities Questionnaire (MLTAQ-Br), which assesses physical, household, social, cultural, and cognitively stimulating leisure activities, were determined, with a median of 3,584.50 minutes per week. The interviewed population was stratified into two groups: Group I — scores >3,584.50 minutes per week (including the median); and Group II — scores ≤3,584.50 minutes per week.

General cognitive functioning was assessed using the Addenbrooke’s Cognitive Examination – Revised (ACE-R)^10^. Mood and anxiety variables were measured using the Geriatric Depression Scale (GDS–15)^11,12^, and the Depression, Anxiety and Stress Scale (DASS-21)^13^. Quality-of-life aspects were assessed using the Control, Autonomy, Self-realization and Pleasure scale (CASP-19)^14,15^. Lastly, life activities were measured using the MLTAQ-Br^16,17^.

### Procedures

The present study is based on a sample from a research project entitled “*Desempenho Cognitivo de idosos e Estilo de Vida Ativo Durante a Pandemia da Covid-19*” (Cognitive Performance of Older Adults and Active Life style during the COVID-19 Pandemic), previously approved by the Human Research Ethics Committee of the Escola de Artes, Ciências e Humanidades (EACH-USP), under permit No. 5.568.787, pursuant to Resolution 466/2012 and 510/2016 governing research ethics involving human participants. The study was also submitted to the Research Committee of the Municipal Secretariat for Social Welfare and Development and received approval on August 8, 2022.

This study was based on a cross-sectional sample of older adults assessed at a single time point in 2022. Information on lifestyle activities was collected retrospectively, referring to the 12 months prior to the interview and spanning different phases of the COVID-19 pandemic.

Following invitation, participants who agreed to take part in the study attended the center at a previously scheduled date and time. All participants read or were read the full Informed Consent together with a staff member (social worker or psychologist from the center). This process culminated in the signing of the document (in duplicate), confirming that participants were aware of the terms described and of their rights, including the right to withdraw from the study at any time if they wished. One copy of the form was retained by the participant and the other by the researcher.

Administration of the data collection instruments took approximately 90 minutes. Assessments were carried out by the researcher. Participant recruitment and interviews took place during the second half of 2022 in a single assessment session.

### Data analysis

To describe the sample profile, frequency tables and descriptive statistics with measures of central tendency and dispersion were constructed. The Shapiro-Wilk test indicated the absence of a normal distribution for most continuous and ordinal variables; therefore, nonparametric tests were applied for statistical inference (Shapiro & Wilk, 1965). The chi-square test was used to compare categorical variables, while the Mann-Whitney U test for independent samples was applied to compare continuous or ordinal variables between groups.

To analyze correlations between cognitive and psychosocial variables and participants’ active lifestyle, Spearman’s correlation test was employed. Based on the division of older adults into two groups, a dichotomous variable was created (Group I = MLTAQ-Br or subdomain scores equal to or above the median; Group II = MLTAQ-Br or subdomain scores below the median). These variables allowed the construction of Table 1.

**Table 1:** Sociodemographic variables of participants overall and stratified by median MLTAQ-Br score (overall score in minutes per week).

Variable	Overall	Group I	Group II	p-value
	n=50	n=25	n=25	
No. of activities performed at center in the last 12 months				0.714^a^
Mean (SD)	3.14 (0.95)	3.16 (0.94)	3.12 (0.97)	
Median (Min.–Max.)	3.00 (1.00–6.00)	3.00 (1.00–6.00)	3.00 (2.00–5.00)	
Time participating in the center (months)				0.514^a^
Mean (SD)	56.92 (50.99)	61.52 (54.29)	52.32 (48.13)	
Median (Min.–Max.)	45.00 (3.00–144.00)	48.00 (4.00–144.00)	36.00 (3.00–144.00)	
MLTAQ-Br (Overall Score)				<0.001^a^
Mean (SD)	3,666.30 (1,443.11)	4,821.84 (1,049.28)	2,510.76 (607.53)	
Median (Min.-Max.)	3,584.50 (1,145–7,410)	4,560.00 (3,645–7,410)	2,400.00 (1,145–3,524)	

Note: ^a^Mann-Whitney U-test.

Abbreviations: MLTAQ-Br: Minnesota Leisure Time Activities Questionnaire – Brazilian Portuguese version; SD: standard deviation.

Finally, as a multivariate technique, logistic regression analysis using the stepwise method was performed to determine which types of activities predicted better performance on the ACE-R (total score) (Goss-Sampson, 2022)^18^. Data were entered into an online Google spreadsheet, and statistical analyses were conducted using JASP, a statistical software program based on R (Goss-Sampson, 2022). The significance level adopted for all statistical tests was 5%, that is, p<0.05.

## RESULTS

A total of 50 older individuals with a mean age of 70.98±5.49 years (range 61-82 years) were assessed. The study participants were predominantly female, widowed, and had a mean educational level of 5.46±3.97 years. The group was stratified according to the median overall score on the MLTAQ-Br (Table 1), revealing a higher self-reported diagnosis of depression and greater involvement in activities for Group I compared with Group II.

The results on the cognitive assessment test and of symptoms of depression, stress, and anxiety, perceived quality of life, together with overall score in minutes per week engaged in physical, domestic, leisure-time, intellectually stimulating, and work-related activities, are presented in Table 2. Group I showed better overall performance and visuospatial ability on the ACE-R. The results showed a statistically significant difference (p<0.05) between Groups I and II in overall performance and visuospatial ability on the ACE-R.

**Table 2:** Performance of participants on tests stratified by the median MLTAQ-Br score (overall score in minutes per week).

Variables	Group	n	Mean	Standard Deviation	Min.	Median	Max.	p-value
ACE-R (Overall Score)	Group I	25	74.20	14.24	33.00	77.00	93.00	0.021
	Group II	25	68.00	11.05	50.00	68.00	95.00	
ACE-R (Visuospatial)	Group I	25	12.64	3.35	4.00	14.00	16.00	0.008
	Group II	25	10.68	2.51	6.00	11.00	15.00	
	Overall	50	11.66	3.09	4.00	12.00	16.00	
GDS (Total Score)	Group I	25	2.84	2.58	0.00	3.00	12.00	0.060
	Group II	25	3.88	2.24	0.00	4.00	8.00	
	Overall	50	3.36	2.45	0.00	3.00	12.00	

Note: p-value for Mann-Whitney U-test

Abbreviations: ACE-R: Addenbrooke’s Cognitive Examination – Revised; GDS: Geriatric Depression Scale.

Performance on the cognitive and psychosocial tests, according to the median for the intellectually stimulating activities variable (minutes per week), is presented in Table 3. The data reveal that the group with greater involvement in this category of activities attained better results on all cognitive abilities assessed (attention, orientation, memory, verbal fluency, language, and visuospatial perception). The same group also scored higher on the autonomy variable.

**Table 3:** Performance of participants on tests stratified by the median MLTAQ-Br score (intellectually stimulating activities in minutes per week).

Variables	Group	n	Mean	Standard Deviation	Min.	Median	Max.	p-value
ACE-R (Overall Score)	Group I	25	76.92	10.78	50.00	77.00	95.00	0.001
	Group II	25	65.28	12.57	33.00	68.00	87.00	
	Overall	50	71.10	13.00	33.00	72.00	95.00	
ACE-R (Attention and Orientation)	Group I	25	14.76	2.26	11.00	15.00	18.00	0.041
	Group II	25	13.40	2.00	8.00	14.00	17.00	
	Overall	50	14.08	2.22	8.00	14.00	18.00	
ACE-R (Memory)	Group I	25	17.84	3.85	10.00	18.00	25.00	0.004
	Group II	25	14.08	4.57	3.00	14.00	22.00	
	Overall	50	15.96	4.59	3.00	16.00	25.00	
ACE-R (Fluency)	Group I	25	9.08	2.55	4.00	9.00	14.00	0.017
	Group II	25	7.32	2.41	2.00	8.00	12.00	
	Overall	50	8.20	2.61	2.00	8.50	14.00	
ACE-R (Language)	Group I	25	22.40	2.87	12.00	23.00	26.00	0.045
	Group II	25	20.00	4.57	7.00	21.00	26.00	
	Overall	50	21.20	3.97	7.00	23.00	26.00	
ACE-R (Visuospatial)	Group I	25	12.84	2.41	8.00	14.00	16.00	0.011
	Group II	25	10.48	3.29	4.00	10.00	16.00	
	Overall	50	11.66	3.09	4.00	12.00	16.00	
CASP-19 (Autonomy)	Group I	25	6.72	1.77	4.00	6.00	10.00	0.020
	Group II	25	5.28	1.51	3.00	6.00	7.00	
	Overall	50	6.00	1.78	3.00	6.00	10.00	

Note: p-value for Mann-Whitney U-test.

Abbreviations: ACE-R: Addenbrooke’s Cognitive Examination – Revised; CASP-19: Control, Autonomy, Self-realization, and Pleasure Scale.

The results of the Depression, Anxiety, and Stress Scale showed statistical significance only for the physical activities variable (minutes per week), as highlighted in Table 4. No statistically significant differences were observed for the stress variable.

**Table 4:** Performance of participants on tests stratified by the median MLTAQ-Br score (physical activity in minutes per week).

Variables	Group	n	Mean	Standard Deviation	Min.	Median	Max.	p-value
DASS21 (Overall Score)	Group I	25	7.60	8.12	00.00	05.00	30.00	0.012
	Group II	25	13.88	11.69	2.00	12.00	46.00	
	Overall	50	10.74	10.46	00.00	07.00	46.00	
DASS21 (Stress)	Group I	25	4.56	3.80	00.00	3.00	13.00	0.239
	Group II	25	6.48	4.94	00.00	6.00	18.00	
	Overall	50	5.52	4.47	00.00	5.00	18.00	
DASS21 (Anxiety)	Group I	25	1.60	2.47	00.00	2.00	13.00	0.013
	Group II	25	3.56	3.51	00.00	1.50	13.00	
	Overall	50	2.58	3.16	00.00	00.00	9.00	
DASS21 (Depression)	Group I	25	1.44	2.62	00.00	00.00	9.00	0.013
	Group II	25	3.84	4.71	00.00	2.00	18.00	
	Overall	50	2.64	3.96	00.00	1.00	18.00	

Note: p-value for Mann-Whitney U-test.

Abbreviation: DASS21: Depression, Anxiety, and Stress Scale.

In order to determine which activity types predicted better performance on the ACE-R (total score), stepwise logistic regression was performed using a series of independent variables. Models were compared using several performance indicators, including deviance, Akaike Information Criteria (AIC), Bayesian Information Criteria (BIC), degrees of freedom (df), log-likelihood difference (ΔX^2^), probability (p), and McFadden R^2^, Nagelkerke R^2^, Tjur R^2^, and Cox & Snell R^2^ tests. Model 4 proved to be the best model, with the lowest deviance (52.896) and the highest McFadden R^2^ (0.236), Nagelkerke R^2^ (0.372), Tjur R^2^ (0.290), and Cox & Snell R^2^ (0.279) values. These indicators suggest that Model 4 better explains the variability in the dependent variable and has the greatest predictive ability. In addition, Model 4 exhibited the lowest AIC (60.896) and BIC (68.544) values, indicating it is the most efficient model. Taken together, these results suggest that Model 4 is the optimal model for explaining cognitive performance based on the independent variables evaluated (Table 5).

**Table 5:** Summary of stepwise logistic regression models.

Model	Deviation	AIC	BIC	df	^∆X^ ^2^	p	McFadden R^2^	Nagelkerke R^2^	Tjur R^2^	Cox & Snell R^2^
1	69,235	71,235	73,147	49			0.000		0.000	
2	60,991	64,991	68,815	48	8,244	0.004	0.119	0.203	0.160	0.152
3	57,957	63,957	69,693	47	3,035	0.082	0.163	0.269	0.213	0.202
4	52,896	60,896	68,544	46	5,061	0.024	0.236	0.372	0.290	0.279

Note: ∆X^2^: Log-likelihood difference.

Abbreviations: AIC: Akaike Information Criteria; BIC: Bayesian Information Criteria; df: Degrees of freedom; p: Probability.

## DISCUSSION

The objective of the present study was to investigate the relationship between active lifestyle and cognitive performance in community-dwelling older adults. For the study, individuals participating in a Community Center for Older Adults in the district of Pedreira, situated in the southern part of the city of São Paulo, were interviewed. Variables measuring cognitive performance, depressive, stress, and anxiety symptoms, and quality of life were assessed, along with engagement in physical, leisure-time and intellectually stimulating activities during the 12 months leading up to data collection.

It is important to highlight that, during the pandemic period, the strictness of health measures imposed on the population varied, with consequent changes in opportunities for social interaction. For example, comparing the situation in Brazil in August 2020, prior to availability of vaccines, with the scenario in August 2022, when the population had been vaccinated, revealed that the frequency of social interactions increased during 2022.

Also, in some areas, guidelines for compulsory use of protective face masks remained in place in 2022. Thus, although there were clearly more possibilities for social interaction after the lifting of stricter measures, the situation had not returned to pre-pandemic levels.

The results revealed that the number of minutes engaged in physical, domestic, leisure-time, intellectually stimulating, and work-related activities was associated with better overall cognitive performance and visuospatial ability. These results corroborate findings in the literature, such as those reported by Sposito, Neri, and Yasuda^19^, showing that community-dwelling older adults with greater engagement in advanced activities of daily living (AADLs) had higher scores on the Mini-Mental State Examination (MMSE).

The longitudinal study by Fu et al.^20^, involving more than 9,000 individuals aged ≥60 years, investigated the association between involvement in social activities and participants’ cognitive function. The researchers assessed orientation, attention, episodic memory, visuospatial abilities, and involvement in physical activity, voluntary work, and cognitively stimulating activities, such as playing board games. Data on sociodemographic and psychosocial variables were also collected. Similar to the present study, the results revealed that greater involvement in activities was associated with better cognitive performance.

Education is one of the main variables influencing the maintenance of cognitive capacity during late life^21,22^. This phenomenon can be explained by the greater cognitive reserve acquired through exposure to stimuli involved in learning, favoring an increase in neural connections and their use to attenuate the effects of cognitive decline^23^.

Another important variable for cognitive reserve is participation in cognitively-stimulating activities^24^. The main characteristics of these activities include low association with physical energy expenditure, little or no interference from social aspects, and a predominance of information processing^25,26^. Analysis of the data collected showed that the two variables (education and engagement in cognitively stimulating activities) were correlated. In this case, the results show that individuals with more years of formal education have greater engagement in cognitively stimulating activities which, in the present investigation, encompassed reading, carrying out manual activities, and playing board games.

Similarly, individuals who had greater involvement in cognitively stimulating activities attained better scores on the cognitive tests, with statistically significant results for the overall score on the ACE-R and all subdomains assessed (attention, orientation, memory, language, fluency, and visuospatial perception). Moreover, participants who engaged more in these activities had a greater sense of autonomy.

Mirroring the findings of the present study, the investigation by Brandebusque et al.^23^ analyzing data from the Frailty in Older Adults Study (Estudo Fragilidade em Idosos Brasileiros – FIBRA) for 2016-2017, found that older individuals aged ≥80 years who performed more cognitively stimulating activities scored higher on the MMSE. In addition, an association was identified with sociodemographic variables such as gender and education.

The literature addressing specific stimuli for the improvement or maintenance of cognitive functioning through cognitive training is extensive. However, there are few studies centered on the benefits of intellectually stimulating activities that do not rely on a set of specific exercises to be performed.

In both public and private services, although interdisciplinary teams (psychologists, social workers, plus workshop instructors in the present study) play a key role, gerontology professionals can add further value to the services provided. The adoption of a gerontological plan, for example, managed by a professional holding a degree in gerontology, may contribute by monitoring the benefits of individuals participating in Community Centers for Older Adults and by investigating individual aspects to be prioritized in order to promote stronger ties, as well as specific gains in the biopsychosocial health of the older adults involved.

Mota^27^, a holder of a degree in gerontology, interviewed health professionals to identify their knowledge regarding the role of gerontologists in the Brazilian Unified Health System (Sistema Único de Saúde – SUS). Some professionals reported being unaware of this qualification and, after obtaining information about gerontology degree holders, felt that they would be important additions to the team, both to assist in caring for older patients and their relatives and to help train the team. In the appropriate context, this focus can also be applied to social services.

Therefore, besides adding to the literature on active lifestyle, cognitive performance, and psychosocial variables in older individuals, the outcomes of the study can help formulate public policies aimed at stimulating actions that promote active aging.

This study has important limitations that should be considered when interpreting the results. First, lifestyle data were obtained through retrospective self-report covering the previous 12 months and may have been subject to recall bias. In addition, data collection was carried out in 2022, a period marked by heterogeneous pandemic conditions, including phases of stricter lockdown measures and the gradual lifting of restrictions. Consequently, the activities reported may reflect a mixture of distinct behavioral contexts, limiting the temporal specificity of the findings.

Additionally, the assessment of lifestyle activities using the MLTAQ-Br is based on quantifying the time spent on different activities (minutes per week), which may fail to adequately capture qualitative aspects such as cognitive complexity, level of engagement, or contextual meaning^28,29^.

Moreover, the study sample was relatively small and drawn from a single community center, with a predominance of women and individuals with low educational level, which may limit the generalizability of the findings. Also, the grouping strategy based on the median MLTAQ-Br scores may have introduced a risk of misclassification bias, given the inherent variability of self-reported measures of activity engagement.

Lastly, although the study aimed to investigate the relationship between lifestyle and cognition in the context of the COVID-19 pandemic, the cross-sectional design limits causal inference. Therefore, the findings should be interpreted as associations contributing to the understanding of the phenomenon and to hypothesis generation for future studies.

In conclusion, taken together, the results of the present study investigating the cognitive performance of the participants, as well as mood, perceived quality of life and active lifestyle in terms of engagement in physical, domestic, leisure-time, cognitively stimulating, and work-related activities, showed that cognitive performance, although strongly correlated with education, was also influenced by engagement in social activities, particularly those of an intellectual nature.

These findings suggest that staying socially active throughout the lifespan and during late life is one of the best ways to attain good physical, mental, and cognitive health, besides strengthening social relationships. Hence, as evidenced by this study, involvement in cognitively stimulating activities was strongly associated with a lower prevalence of psychosocial symptoms involving depression, anxiety, and stress, a better perception of qualify-of-life aspects, such as autonomy, and better cognitive performance regarding the functions of memory, attention, language, fluency, and visuospatial perception.

Involvement in activities aimed at the older population favors engagement in social activities and, consequently, the maintenance of an active lifestyle. In the case of the older adults interviewed, whose sociodemographic characteristics are associated with greater social vulnerability (*e.g*., low income and few years of formal education), actions promoting this engagement, such as the service offered by Community Centers for Older Adults, are paramount.

In this respect, amid situations such as those experienced during the COVID-19 pandemic, involving the need for social distancing, isolation, and lockdowns, it is important to devise measures that ensure older individuals are not deprived of opportunities to remain socially active, thereby increasing the chances of maintaining cognitive performance rather than experiencing expected cognitive decline, a process driven by intrinsic and extrinsic factors.

Future studies investigating this subject should involve samples that are more sociodemographically heterogeneous, allowing the association between lifestyle and cognitive performance to be determined in individuals with different income levels, years of education, and degrees of participation in work-related activities, for example. Further investigations could also explore more qualitative data collected through open-ended questions.

## Data Availability

The datasets generated and/or analyzed during the current study are available from the corresponding author upon reasonable request.
